# Root Canal Morphology of Premolars in Saudis

**DOI:** 10.7759/cureus.45888

**Published:** 2023-09-25

**Authors:** Mahir A Mirah, Arwa Bafail, Abdulmajeed Baik, Basim Abu zaid, Muhannad Hakeem, Hani Ghabbani

**Affiliations:** 1 Restorative Dental Sciences Department, College of Dentistry, Taibah University, Madinah, SAU; 2 Internship Program, College of Dentistry, Taibah University, Madinah, SAU; 3 Endodontic Department, King Faisal Specialist Hospital and Research Centre, Madinah, SAU

**Keywords:** mandibular premolars, maxillary premolars, root number, canal configuration, saudi arabia, cone-beam computed tomography

## Abstract

Aim: This study aimed to evaluate root number and morphological variations of the root canal system in maxillary and mandibular premolars among the Saudi subpopulation.

Methodology: A total of 500 cone-beam computed tomography (CBCT) images were assessed, including 2442 maxillary and mandibular premolars. The evaluation encompassed the number of roots and canals and their configuration based on the Vertucci classification. Gender differences were considered, along with the position of premolars and unilateral or bilateral symmetry.

Results: Maxillary first premolars predominantly exhibited two roots (82.6%), followed by one root (16.3%) and three roots (0.97%), with a majority having two root canals (83.6%). Regarding maxillary second premolars, the most common morphology was one root (66.6%), with two root canals (40.5%) observed more frequently than one root canal (38.1%). Mandibular first and second premolars mainly featured one root (84.8% and 96.1%, respectively) with one root canal (70.8% and 90.2%, respectively), whereas two root canals were less prevalent (2.3% and 1.8%, respectively). Three canals and three roots were rarely found in mandibular premolars (0.3%). Regarding maxillary premolars, males exhibited significantly higher type IV and mandibular type I root configurations compared with females. Conversely, regarding mandibular premolars, females showed significant mandibular type V and higher type I root configurations compared with males.

Conclusion: CBCT imaging facilitated precise assessment of root morphology and root canal configurations in maxillary and mandibular premolars. The present findings can aid dentists in diagnosing, evaluating case difficulty, and devising effective root canal treatments, particularly in patients from Saudi Arabia.

## Introduction

Bacterial infection is the primary cause of pulpal and periapical disease [1]. Root canal therapy aims to prevent reinfection and effectively eliminate or significantly reduce bacterial load and byproducts from the root canal system. Treatment failure may result from missed canals, incorrect working length, incomplete root canal obturation, suboptimal disinfection of the root canal system, and anatomical variations [2]. Understanding the morphological variations of root canal anatomy is critical during root canal treatment [3]. Therefore, clinicians require proper knowledge of root canal morphology to employ the most suitable treatment approach and improve outcomes [4]. Morphological variations in the root canal system play a crucial role in endodontic therapy; missed canals may lead to the persistence of necrotic tissues and microbes within the root canal anatomy, resulting in periapical lesions [5]. The difficulty encountered during root canal therapy on premolar teeth stems from anatomical variances in canal configurations and root count [6]. Various classification systems, including Vertucci, Weine, and Gulabivala, have been used to understand the root canal system morphology [7-9]. Vertucci's classification is most commonly used for describing root canal system configuration, which consists of eight types. Further investigation such as different types of radiographs to assess tooth anatomy and identify difficulties is essential before starting endodontic treatment.

Radiographs are essential tools for diagnosing and investigating anatomical variations of the root canal system. Two- and three-dimensional imaging are the most commonly used radiographs in endodontic diagnosis and treatment. However, two-dimensional imaging has limitations in detecting the number of roots, obstacles that can alter or cover the root canal, and canal or root curvature. Cone-beam computed tomography (CBCT) is a valuable technique that provides accurate three-dimensional representations of tooth anatomy internally and externally without causing destruction. Moreover, CBCT has a high accuracy rate in terms of assessing the complexity of root canal anatomy [10]. It can be used to evaluate root canal configuration with an accuracy comparable to clearing and staining methods, which were previously considered more reliable for studying root canal anatomy owing to their ability to offer precise three-dimensional details of tooth morphology [5]. However, radiation exposure remains a significant concern, particularly for repeated or unnecessary scans. Unlike traditional two-dimensional dental X-rays, CBCT exposes patients to a higher radiation dose due to the increased complexity and coverage of the imaging process [11]. The potential risks associated with using CBCT on pediatric patients due to their developing organs and tissues and on pregnant women must be considered, particularly with regard to possible exposure of the developing fetus to radiation [12]. Genetic considerations and cumulative radiation dose on the patients should be considered [13]. The clinician should be aware of these risks and follow the ALARA (As Low As Reasonably Achievable) principle to ensure the lowest possible radiation dose is used while still achieving diagnostic objectives [14].

A previous CBCT study conducted on a Spanish population evaluated morphological variations in maxillary premolars and found that two roots were the most frequently observed (n=221; 51.4%), with type IV canal configuration being prevalent in the majority of teeth (n=227; 52.8%) [15]. However, in maxillary second premolars, one root was predominant (n=310; 82.9%), with type I canal configuration observed in most cases (n=147; 47.2%) [15]. Another study in a Chinese population assessed morphological variations in maxillary premolars and reported that 45 (33%) maxillary first premolars had two roots, and 153 (51%) had type IV canal configuration [16]. A systematic review investigating the morphological variations of mandibular premolars in the Iranian population reported a high prevalence of multiple root canals (17-29%) [17].

Understanding the complex three-dimensional structure of root canal anatomy and its possible diversifications is crucial for achieving optimal treatment results. Considering anatomical variations of root canal morphology can markedly reduce complications that may arise during access cavity preparation, cleaning, shaping, and filling. The literature includes multiple studies that discuss and describe the morphological structure of root canal anatomy [3,8,18].

This study aimed to use CBCT to observe morphological variations of root canals in maxillary and mandibular premolars in a Saudi subpopulation. Therefore, this research provides a theoretical reference for clinical treatments.

## Materials and methods

This cross-sectional study analyzed CBCT images obtained from the College of Dentistry, Taibah University, Saudi Arabia, collected between January 2019 and June 2022. The research protocol received ethical approval from the ethics committee of the College of Dentistry, Taibah University (protocol no. TUCDREC/051022/ASBafail). Reporting of the current cross-sectional study adhered to the Strengthening the Reporting of Observational Studies in Epidemiology (STROBE) statement guidelines [19].

**Figure 1:**
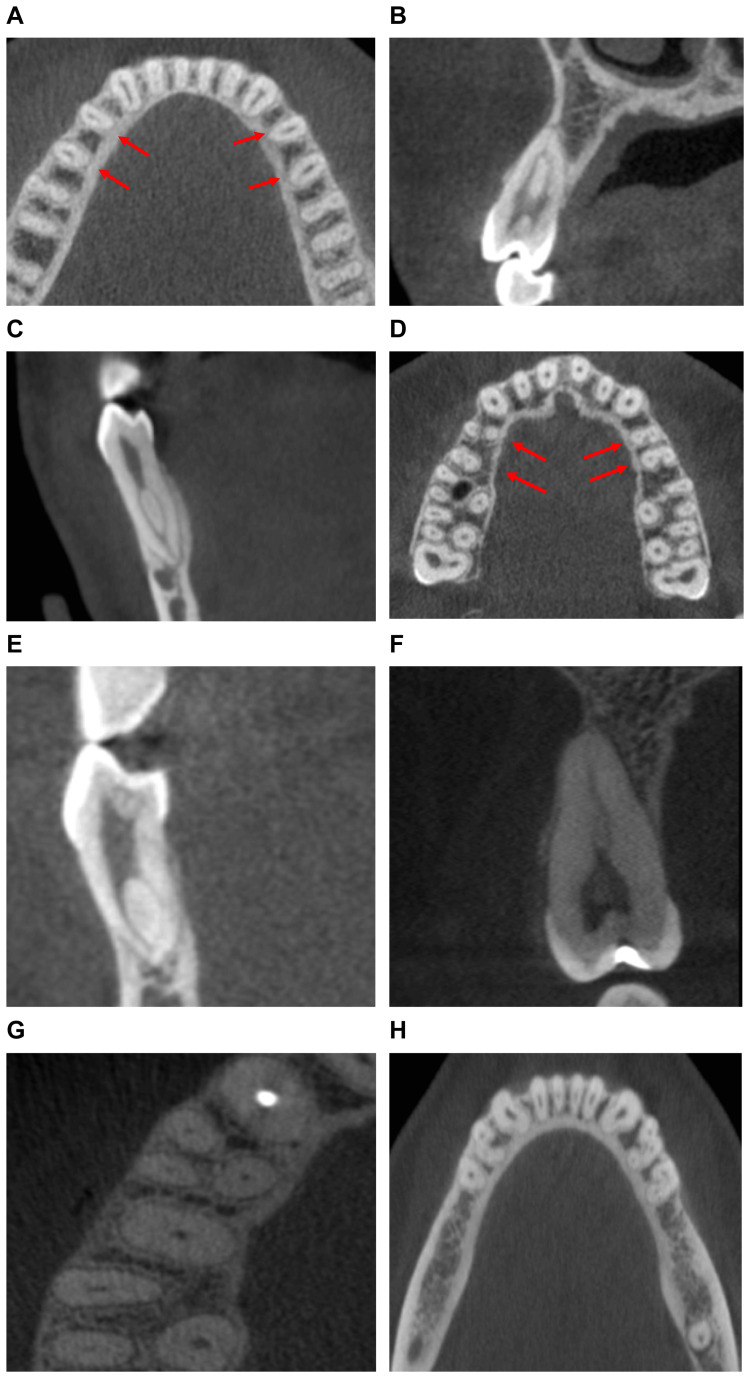
Root canal configuration of premolar teeth shown in CBCT images (A) Axial view showing type I canal configuration; (B) Coronal view showing type II canal configuration; (C) Coronal view showing type III canal configuration; (D) Axial view showing type IV canal configuration; (E) Coronal view showing type V canal configuration; (F) Coronal view showing type VII canal configuration; and (G, H) Axial views showing type VIII canal configuration. CBCT: cone-beam computed tomography

Following Vertucci's classification, CBCT images with axial and coronal views were used to observe and record the canal configuration and counts of roots in maxillary and mandibular premolars (Figure 1).

The CBCT images were examined by two consultant endodontists. An interexaminer reliability test showed a 95% agreement between the two observers in their assessment of 10 CBCT images.

The sample size of this study included 500 CBCT images of Saudi patients (250 females; 250 males) aged 18-75 years, selected through a random sampling process. These patients required oral and maxillofacial treatment for various purposes, including implant placement, missed canal, or dental surgery, and were referred to the Radiology Department at the College of Dentistry, Taibah University, between 2019 and 2022. CBCT images of maxillary and mandibular premolars with fully formed roots were considered. Exclusion criteria encompassed distorted or unclear images, periapical lesions, teeth with crowns or posts, previously root canal-treated teeth, and teeth with pathological or physiological conditions, e.g., incomplete root formation.

CBCT images were acquired using the Carestream CS 9300 system (Carestream Dental LLC, Atlanta, Georgia, United States) at the Radiology Department of the College of Dentistry, Taibah University. The scan parameters based on patient body size were used to obtain high-quality images for precise patient positioning during radiation exposure and reduce radiation dose to the patients with no negative impact on image quality. The machine parameters for medium patients were set at 90 kVp, 4 mA, and 8 s acquisition time, whereas for large patients, the settings were 90 kVp, 5 mA, and 8 s acquisition time.

Data were transferred from an Excel spreadsheet (Microsoft Corporation, Redmond, Washington, United States) to IBM SPSS Statistics for Windows, Version 26.0 (Released 2019; IBM Corp., Armonk, New York, United States) for analysis. Descriptive analyses were conducted to report sample characteristics, with quantitative variables (number of roots and number of each canal configuration) analyzed as frequency and percentages. A chi-square test was used to compare gender subgroups and the number of roots and root canal configurations. The level of significance was set at p ≤ 0.05.

## Results

From 500 patients (250 males; 250 females), 2442 premolars were evaluated, consisting of 613 maxillary first premolars, 566 maxillary second premolars, 663 mandibular first premolars, and 600 mandibular second premolars. A summary of the number of roots in maxillary and mandibular premolars of males and females is found in Table 1.

**Table 1:** Number of roots of maxillary and mandibular premolars The total number of teeth is 2442, divided into 1232 teeth for males and 1210 teeth for females n = the number of teeth; ^*^ = statistically significant difference between males and females (P < 0.05); T = total; M = males; F = females

	One Root			Two Roots			Three Roots			
Tooth # (n)	M, n (%)	F, n (%)	T, n (%)	M, n (%)	F, n (%)	T, n (%)	M, n (%)	F, n (%)	T, n (%)	P-Value
#14 (316)	11 (7.30%)	40 (24.20%)	51 (15.75%)	138 (91.40%)	124 (75.20%)	262 (83.30%)	2 (1.30%)	1 (0.60%)	3 (0.95%)	0.00^*^
#15 (283)	86 (64.20%)	105 (70.50%)	191 (67.35%)	47 (35.10%)	44 (29.50%)	91 (32.30%)	1 (0.70%)	0 (0.00%)	1 (0.35%)	0.33
#24 (297)	12 (8.30%)	39 (25.70%)	51 (17.00%)	132 (91.00%)	111 (73.00%)	243 (82.00%)	1 (0.70%)	2 (1.30%)	3 (1.00%)	0.00^*^
#25 (283)	85 (63.90%)	102 (68.00%)	187 (65.95%)	48 (36.10%)	47 (31.30%)	95 (33.7%)	0 (0.00%)	1 (0.70%)	1 (0.35%)	0.46
#34 (335)	145 (83.30%)	139 (86.30%)	284 (84.80%)	29 (16.70%)	21 (13.00%)	50 (14.85%)	0 (0.00%)	1 (0.60%)	1 (0.30%)	0.39
#35 (304)	150 (93.80%)	142 (98.60%)	292 (96.20%)	10 (6.30%)	2 (1.40%)	12 (3.85%)	0 (0.00%)	0 (0.00%)	0 (0.00%)	0.03^*^
#44 (328)	145 (83.30%)	133 (86.40%)	278 (84.85%)	29 (16.70%)	20 (13.00%)	49 (14.85%)	0 (0.00%)	1 (0.60%)	1 (0.60%)	0.38
#45 (296)	151 (93.80%)	133 (98.50%)	284 (96.15%)	10 (6.20%)	2 (1.50%)	12 (3.85%)	0 (0.00%)	0 (0.00%)	0 (0.00%)	0.40
T (2442)	1618 (66.26%)			814 (33.33%)			10 (0.41%)			

In male patients, 296 and 267 of maxillary first and second premolars were evaluated, with the highest observed root morphology being two roots (n=270; 91.20%) and one root (n=171; 64.04%), respectively. In addition, 348 and 321 mandibular first and second premolars were evaluated, with the most common root morphology being one root (n=290; 83.30% and n=301; 93.80%, respectively). Among females, 317 and 299 maxillary first and second premolars were evaluated, with the most frequent root anatomy being two roots (n=235; 74.10%) and one root (n=207; 69.25%), respectively. Regarding mandibular first and second premolars, 315 and 279 teeth were assessed, with the most frequent root morphology being one root (n=272; 86.35%) and (n=275; 98.57%), respectively. Significant differences were found between genders in terms of the number of roots of maxillary right premolars, maxillary left premolars, and mandibular left second premolars (P = 0.000, P = 0.000, and P = 0.030, respectively). No significant differences were observed for other tooth types (P > 0.05).

The root canal configuration of maxillary and mandibular premolars in males and females was summarized according to Vertucci's classification in Table 2.

**Table 2:** Root canal configurations of maxillary and mandibular premolars The total number of teeth is 2442, divided into 1232 teeth for males and 1210 teeth for females. n = the number of teeth; * = statistically significant difference between males and females (P < 0.05); T = total; M = males; F = females

Tooth No. (n)	Type I			Type II			Type III			Type IV			Type V			Type VI			Type VII			Type VIII			P-value
	M, n (%)	F, n (%)	T, n (%)	M, n (%)	F, n (%)	T, n (%)	M, n (%)	F, n (%)	T, n (%)	M, n (%)	F, n (%)	T, n (%)	M, n (%)	F, n (%)	T, n (%)	M, n (%)	F, n (%)	T, n (%)	M, n (%)	F, n (%)	T, n (%)	M, n (%)	F, n (%)	T n (%)	
#14 (316)	1 (0.7%)	5 (3.0%)	6 (1.85%)	6 (4.0%)	34 (20.6%)	40 (12.3%)	0 (0.0%)	0 (0.0%)	0 (0.0%)	141 (93.4%)	123 (74.5%)	264 (83.95%)	1 (0.7%)	2 (1.2%)	3 (0.95%)	0 (0.0%)	0 (0.0%)	0 (0.0%)	0 (0.0%)	0 (0.0%)	0 (0.0%)	2 (1.3%)	1 (0.6%)	3 (0.95%)	0.00^*^
#15 (283)	40 (29.9%)	71 (47.7%)	111 (38.8%)	18 (13.4%)	27 (18.1%)	45 (15.75%)	1 (0.7%)	4 (2.7%)	5 (1.7%)	67 (50.0%)	47 (31.5%)	114 (40.75%)	7 (5.2%)	0 (0.0%)	7 (2.6%)	0 (0.0%)	0 (0.0%)	0 (0.0%)	0 (0.0%)	0 (0.0%)	0 (0.0%)	1 (0.7%)	0 (0.0%)	1 (0.35%)	0.00^*^
#24 (297)	1 (0.7%)	8 (5.3%)	9 (3%)	7 (4.8%)	31 (20.4%)	38 (12.6%)	0 (0.0%)	0 (0.0%)	0 (0.0%)	136 (93.8%)	111 (73.0%)	247 (83.4%)	0 (0.0%)	0 (0.0%)	0 (0.0%)	0 (0.0%)	0 (0.0%)	0 (0.0%)	0 (0.0%)	0 (0.0%)	0 (0.0%)	1 (0.7%)	2 (1.3%)	3 (1%)	0.00^*^
#25 (283)	39 (29.3%)	68 (45.3%)	107 (37.3%)	22 (16.5%)	25 (16.7%)	47 (16.6%)	0 (0.0%)	4 (2.7%)	4 (1.35%)	64 (48.1%)	49 (32.7%)	113 (40.4%)	8 (6.0%)	2 (1.3%)	10 (3.65%)	0 (0.0%)	0 (0.0%)	0 (0.0%)	0 (0.0%)	1 (0.7%)	1 (0.35%)	0 (0.0%)	1 (0.7%)	1 (0.35%)	0.00^*^
#34 (335)	107 (61.5%)	128 (79.5%)	235 (70.5%)	0 (0.0%)	1 (0.6%)	1 (0.3%)	0 (0.0%)	8 (5.0%)	8 (2.5%)	1 (0.6%)	6 (3.7%)	7 (2.15%)	66 (37.9%)	17 (10.6%)	83 (24.24%)	0 (0.0%)	0 (0.0%)	0 (0.0%)	0 (0.0%)	0 (0.0%)	0 (0.0%)	0 (0.0%)	1 (0.6%)	1 (0.3%)	0.00^*^
#35 (304)	134 (83.8%)	137 (95.1%)	271 (89.45%)	2 (1.3%)	2 (1.4%)	4 (1.35%)	1 (0.6%)	2 (1.4%)	3 (1.0%)	0 (0.0%)	2 (1.4%)	2 (0.7%)	23 (14.4%)	1 (0.7%)	24 (7.55%)	0 (0.0%)	0 (0.0%)	0 (0.0%)	0 (0.0%)	0 (0.0%)	0 (0.0%)	0 (0.0%)	0 (0.0%)	0 (0.0%)	0.00^*^
#44 (328)	108 (62.1%)	123 (79.9%)	231 (71%)	0 (0.0%)	1 (0.6%)	1 (0.3%)	0 (0.0%)	7 (4.5%)	7 (2.25%)	1 (0.6%)	5 (3.2%)	6 (1.9%)	65 (37.4%)	17 (11.0%)	82 (24.2%)	0 (0.0%)	0 (0.0%)	0 (0.0%)	0 (0.0%)	0 (0.0%)	0 (0.0%)	0 (0.0%)	1 (0.6%)	1 (0.3%)	0.00^*^
#45 (296)	139 (86.3%)	129 (95.6%)	268 (90%)	0 (0.0%)	2 (1.5%)	2 (0.75%)	0 (0.0%)	2 (1.5%)	2 (0.75%)	0 (0.0%)	2 (1.5%)	2 (0.75%)	22 (13.7%)	0 (0.0%)	22 (6.85%)	0 (0.0%)	0 (0.0%)	0 (0.0%)	0 (0.0%)	0 (0.0%)	0 (0.0%)	0 (0.0%)	0 (0.0%)	0 (0.0%)	0.00^*^
T (2442)	1238 (50.70%)			178 (7.29%)			29 (1.19%)			755 (30.92%)			231 (9.46%)			0 (0.00%)			1 (0.04%)			10 (0.04%)			

In males, Type IV root canal configurations were the most common in maxillary first premolars, 277 (93.60%) teeth, and in maxillary second premolars, 131 (49.05%) teeth. The most common configuration for mandibular first and second premolars was type I (n=215; 61.78% and n=173; 85.00%, respectively. In females, type IV root canal configurations were the most frequent in maxillary first premolars (n=234; 73.81%); however, for maxillary second premolars, type I root canal configurations were the most common (n=96; 46.50%). Regarding mandibular first and second premolars, type I root canal configurations were the most frequent (n=251; 79.70% and n=266; 95.35%, respectively). Significant differences were found between genders in terms of the root configurations of maxillary and mandibular premolars (P<0.05).

Both maxillary and mandibular premolars exhibited a high degree of bilateral symmetry. Males showed a significantly higher rate of type IV canal configuration in maxillary premolars and type I canal configuration in mandibular premolars compared to females, whereas females showed a significantly higher rate of type V canal configuration in mandibular premolars and higher type I canal configuration compared to males (P < 0.05).

## Discussion

This study used CBCT images with different views to investigate morphological variations of root canal anatomy in the Saudi subpopulation's premolars of both the maxillary and mandibular arches. CBCT scan parameters were used according to the patient's body size. Selection of adequate radiation exposure can increase the quality of the images without unnecessarily increasing the radiation dose, which follows the ALARA principle [20].

Compared with previous studies based on the Saudi population, this study can be considered more comprehensive, as it evaluated premolars in both arches, whereas previous studies focused on either maxillary or mandibular premolars with smaller sample sizes [21-23], and these differences may account for the anatomical variations observed among the studies. The Saudi subpopulation exhibits a wide variety of root numbers and canal morphological variations in premolars of the maxillary and mandibular arches. Therefore, it is crucial for clinicians to identify where root canals join and separate to treat all canals effectively.

In previous studies, the percentage ranges of maxillary first premolars with one root, two roots, and three roots were 18.2-37%, 57-80.2%, and 1.33-1.6%, respectively [24,25]. For instance, a study conducted on the Spanish population revealed that the frequency of one-, two-, and three-rooted teeth was 198 (46.0%), 221 (51.4%), and 11 (2.6%), respectively [15]. Another study on the Egyptian subpopulation revealed that the frequency of maxillary first premolars with two roots was 190 (53.1%), which is lower than our finding [26]. Such variations among these studies may be attributed to different geographic areas and sample sizes.

Regarding maxillary second premolars, prior studies found one-, two-, and three-rooted premolars at frequencies of 71.2-82.9%, 15.5-28.4%, and 0.4-6%, respectively [15,24]. The present findings were consistent with one study conducted in Saudi Arabia, where one root was observed in 84 (76.4%) of teeth, two roots in 26 (23.6%), and three roots were not observed [27]. Our findings also aligned with another study conducted on the Saudi subpopulation, in which maxillary second premolars with one root, two roots, and three roots were observed in 416 (83.2%), 79 (15.8%), and five (1.0%) teeth, respectively [28].

Regarding mandibular first premolars, the majority of cases had one root, followed by two roots, and then three roots. These results were similar to those in other studies conducted on the Saudi and Iranian populations [22,29], except for the prevalence of two roots, which was higher in our study, possibly due to differences in geographic areas. Our results also aligned with a study conducted on the South African population, in which mandibular first premolars with one root, two roots, and three roots were found in 378 (97.90%), seven (1.80%), and one (0.25%) teeth, respectively [30]. However, the prevalence of the two roots was much higher in our study, likely due to differences in ethnic background and geographic location.

In the current study, the most common canal configuration in mandibular first premolars was type I, followed by type V, type III, type IV, type II, and type VIII. A study conducted on the Indian population found the type II configuration in two (5%) of the teeth [31]. These variations may be attributed to differences in evaluation criteria and a higher sample size in our study. Ethnicity also seems to have an effect on the morphology of root canal anatomy. The prevalence of type II canal configuration in the Saudi population was previously reported as 14 (3.6%) [22], which is higher than our study, possibly due to regional differences. However, another study on the Saudi population found type I to be the most common canal configuration in the mandibular first premolar 69 (69%) teeth [23], which is consistent with our findings.

Regarding mandibular second premolars, the most prevalent root morphology was one root, followed by two roots. Our findings were consistent with other studies conducted on the South African and Thai populations [30,32].

We found that type I was the most common canal configuration, followed by type V, II, III, and IV. These results were in line with another study conducted on the Turkish population, reporting type I canal configurations in 668 (95.4%) mandibular second premolars [33]. In another study on the German population, one root was the most prevalent in mandibular second premolars 859 (98.6%) teeth, with the type I configuration found in 340 (39%) teeth [34], which aligns with the current findings.

These variations in the anatomy of the root canal system highlight the impact of ethnic background on the morphology of premolar roots in both the maxillary and mandibular arches. Moreover, the CBCT used in this research had a mandibular spatial resolution than nano- and micro-CT, which could have influenced the results.

A limitation of this study is that the sample was taken from the western region of Saudi Arabia, and the findings may only be applicable to part of the Saudi population. In addition, the anatomy of root canals was classified into eight types according to Vertucci's classification without considering additional subcategories. However, applying numerous new subcategories of Vertucci's classification may have limited clinical significance, as root canal preparation during endodontic therapy usually focuses on the main canals.

## Conclusions

The ethnicity of the Saudi subpopulation is widely diverse and includes almost all ethnic backgrounds. A comprehensive understanding of the internal configuration of root canal anatomy and its variations is essential to enhance root canal therapy and minimize procedural errors. CBCT imaging precisely assesses the root morphology and canal configuration in both maxillary and mandibular premolars. In this study, most of the maxillary premolars had two roots, while most mandibular premolars had one root. The type IV root canal configuration was most common in maxillary premolars while the most common root canal configuration in mandibular premolars was type I. Such findings can be instrumental for dentists in diagnosing and evaluating case difficulty and facilitating effective treatment of root canals.
